# Deep vein thrombosis in patients with patellar fractures: Assessing incidence rates and identifying risk factors

**DOI:** 10.1371/journal.pone.0316628

**Published:** 2025-02-21

**Authors:** Shuo Yang, Erdong Zhang, Ziping Li, Yubin Long, Yiran Li, Jiaqi Zhang, Fei Wang, Lin Liu, Tao Wang, Junfei Guo, Zhiyong Hou

**Affiliations:** 1 Orthopaedic Research Institute of Hebei Province, Shijiazhuang, Hebei, P.R. China; 2 The Third Department of Orthopedics, Baoding First Central Hospital, Baoding, Hebei, P.R. China; 3 Department of Anesthesiology, The Second Hospital of Hebei Medical University, Shijiazhuang, Hebei, P.R. China; 4 Department of Orthopaedic Surgery, The Third Hospital of Hebei Medical University, Shijiazhuang, Hebei, P.R. China; 5 Department of Joint Surgery, Honghui Hospital, Xi’an Jiaotong University, Xi’an, China; 6 NHC Key Laboratory of Intelligent Orthopaedic Equipment (The Third Hospital of Hebei Medical University), Shijiazhuang, Hebei, P.R. China; The Fourth Affiliated Hospital Zhejiang University School of Medicine, CHINA

## Abstract

**Background:**

Deep Vein Thrombosis (DVT) represents a significant complication following orthopedic injuries, particularly patellar fractures. Despite the prevalence, comprehensive studies assessing the incidence rates and identifying specific risk factors in patellar fracture patients are limited.

**Methods:**

This retrospective analysis reviewed electronic medical records from 3311 patients treated for patellar fractures at two tertiary hospitals between November 2013 and January 2023. The study focused on patient demographics, fracture characteristics, comorbidities, and laboratory parameters to evaluate the incidence and predictors of DVT. DVT prophylaxis measures and diagnostic criteria, including Doppler Ultrasound Scans, were rigorously applied.

**Results:**

In patients with patellar fractures, the DVT incidence was 30.8%, with 1,790 clots identified in 1,021 diagnosed individuals, predominantly on the injured side (96.7%), and a minor portion on the uninjured side (3.2%). Key risk factors included older age (P<0.001, OR = 1.038), the presence of open injuries (P = 0.002, OR = 1.521), multiple injuries (P<0.001, OR = 3.623), and prolonged time from injury to surgical treatment (P<0.001, OR = 1.097). Conversely, higher levels of albumin (ALB) (P = 0.029, OR = 0.983) and sodium (Na) (P = 0.028, OR = 0.971) were identified as protective factors against DVT. Besides, ROC curve analysis revealed that the age of 52 years and a duration of 4 days from injury to surgery serve as predictive cut-off values for assessing the risk of DVT.

**Conclusion:**

Our study investigates the incidence of thrombosis in patellar fracture patients and identifies key risk factors for DVT, including age, open and multiple injuries, and the time from injury to surgery. Additionally, we highlight sodium and albumin levels as protective factors. By establishing threshold values for age and surgical delay, our findings improve DVT risk assessment, facilitating earlier and more targeted interventions.

## Background

In the realm of adult orthopedic injuries, fractures involving the patella, specifically classified as intra-articular types, constitute a small yet noteworthy proportion, accounting for approximately 2% of all fracture cases [1, 2]. Although occurrences are relatively rare, incidents impacting the knee joint can lead to significant adverse effects. The etiology of patellar fractures typically involves direct trauma to the knee, such as from a fall or collision, or can occur indirectly through abrupt and intense contractions of the quadriceps muscle, which may precipitate a fracture even in the absence of direct impact [3]. For non-displaced fractures, conservative treatments like immobilization may suffice, but most cases require surgical intervention, which, along with comprehensive rehabilitation, typically leads to satisfactory outcomes [4]. However, such fractures significantly limit lower extremity movement, leading to immobilization that, in turn, reduces venous blood flow and increases the risk of deep vein thrombosis (DVT) [5]. This risk is further compounded by the hypercoagulability induced by the inflammatory response to the fracture [6].

DVT is notably common among patients hospitalized for lower extremity fractures, driven by a triad of hypercoagulability, vascular injury, and reduced blood flow velocity [6]. In patellar fracture cases, these risks are compounded by specific factors such as the limited knee mobility during recovery, surgical interventions that can damage local blood vessels, and the prolonged immobilization period. Without timely prevention or intervention, DVT can escalate into chronic pain, trigger the formation of secondary varicose veins, lead to ulcer development, and, in severe cases, cause life-threatening pulmonary embolism (PE). This not only compromises patient outcomes but also adversely impacts their quality of life [7, 8]. This underscores the critical need for early DVT prevention and highlights the importance of proactive risk mitigation.

Researchers have delved into the incidence and predictors of DVT in patients undergoing orthopedic surgeries, with findings from previous studies indicating that the occurrence of DVT varies widely, ranging from 8% to 34.9% [9]. Certain factors, such as male gender, elevated D-dimer levels (>0.5 mg/L), high total cholesterol levels (>5.6 mmol/L), and an elevated platelet-to-lymphocyte ratio (PLR > 189.8), have been identified as significant DVT risks in patients with closed patella fractures [10–12]. However, previous studies primarily focused on the preoperative and postoperative stages, often overlooking the comprehensive analysis afforded by considering the entire duration of a patient’s hospital stay. Our research seeks to rectify this by examining the prevalence and determinants of DVT across the entire hospitalization of individuals with patellar fractures. Building on prior knowledge, our study advocates for an expansive, multicentric approach that not only embraces a larger cohort but also conducts an exhaustive evaluation of all initial laboratory markers. This strategic methodology is designed to bridge existing research gaps, thereby enriching our understanding of DVT risk factors in the context of patellar fractures.

Therefore, our objective is to conduct a more comprehensive retrospective analysis of the incidence and risk factors of DVT following patellar fractures, with the aim of enhancing the understanding of its etiology, refining preventive strategies, and ultimately improving patient outcomes by mitigating potential complications.

## Materials and methods

### Ethics statement

In this study, we analyzed the electronic medical records of patients treated for patellar fractures at two hospitals from November 2013 to January 2023. The data were accessed for research purposes on September 1, 2023. This research received approval from the institutional review boards of the two hospitals, in accordance with the ethical standards set forth in the 1964 Declaration of Helsinki (approval number: 2023-002-1, 2024181). Due to the retrospective nature of the research, the requirement for informed consent from patients was waived by the ethics committee. Informed consent was obtained from all participants in the form of written consent. Given the retrospective nature of this investigation, the requirement for informed consent from patients was waived by the ethics committee. During the data collection period, the authors did not have access to any information that could identify individual participants.

### Patients

This retrospective study was conducted at Hebei Medical University Third Hospital and Baoding No.1 Central Hospital, both recognized as tertiary hospitals equipped with Level I trauma centers.

**Fig 1:**
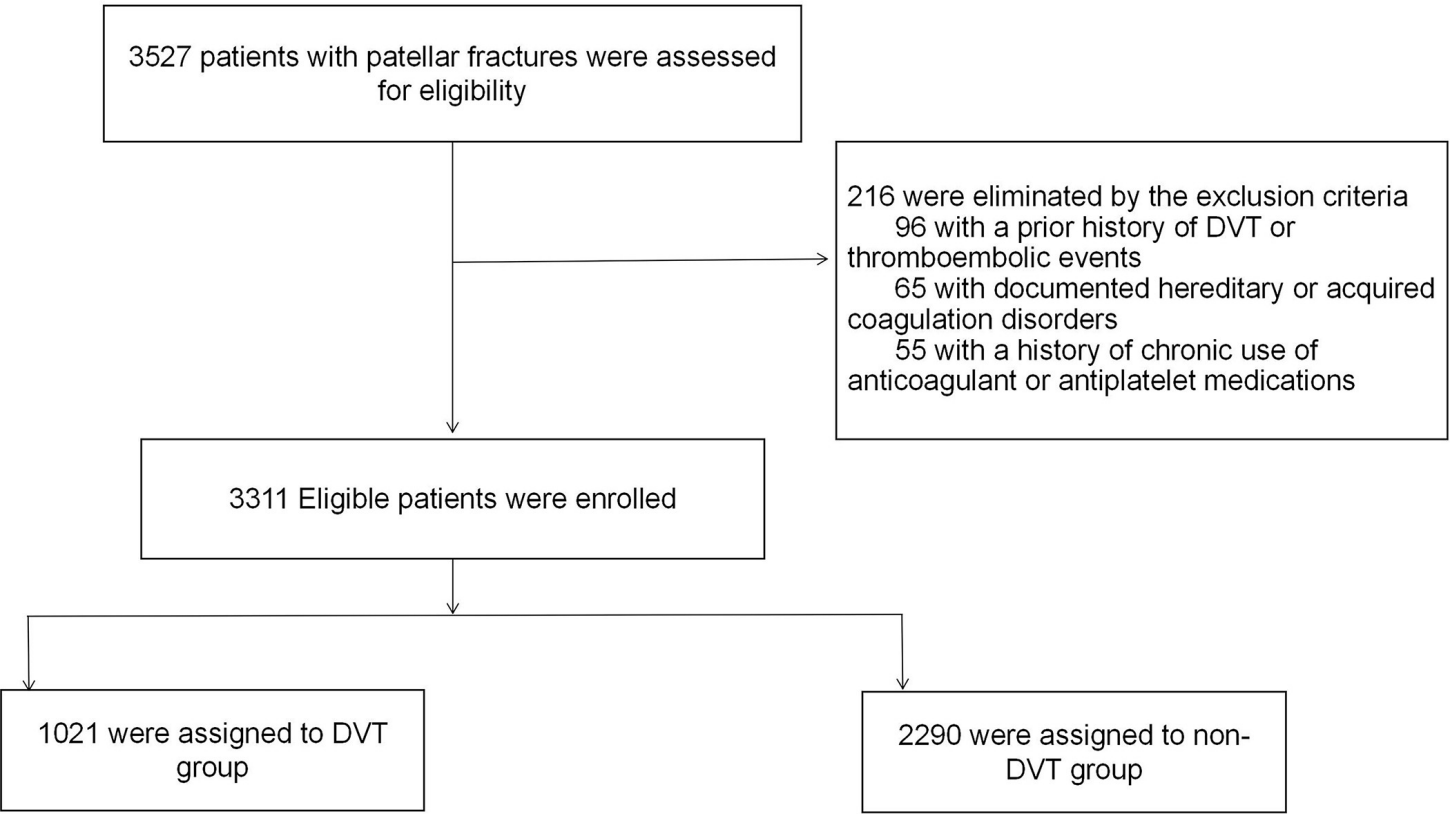
Exclusion criteria and the eligible cases included in this study.

Patients eligible for inclusion in the study were those who: 1.Were aged 18 years and older. 2.Had complete electronic medical records accessible for review. 3.Had been definitively diagnosed with patellar fractures, as verified by radiological imaging techniques. Conversely, the study excluded patients with: 1.A prior history of DVT or thromboembolic events. 2.Documented hereditary or acquired coagulation disorders. 3.A history of chronic use of anticoagulant or antiplatelet medications (Fig 1). Using these criteria, we enrolled 3,311 patients (2,201 men and 1,110 women), dividing them into DVT and non-DVT groups based on diagnosis.

DVT is classified into three distinct categories based on their unique characteristics: proximal DVT, distal DVT, and the mixed type. Proximal DVT is identified by thrombosis formation in the veins at or above the popliteal vein, including the iliac, superficial femoral, femoral, and popliteal veins. In contrast, distal DVT is characterized by thrombosis occurring below the knee, affecting veins such as the tibial, muscular, and peroneal veins. The mixed type, as suggested by its name, represents a combination of both proximal and distal thrombosis.

Throughout their hospitalization, patients were proactively treated to prevent DVT using low-molecular-weight heparin sodium (LMWH) injections to minimize the risk of clot formation. The standard procedure involved administering LMWH, at a dose of 4250 IU daily, from the moment of admission up to 12 hours before surgery, and then continuing 12 hours post-surgery until discharge. For patients identified with DVT, the regimen was intensified to 4250 IU of LMWH sodium twice daily. Additionally, to further mitigate the risk of DVT, a pneumatic compression device was used intermittently for mechanical prophylaxis during their stay.

Upon admission, all participants underwent Doppler Ultrasound Scans (DUS) of both lower extremities, with follow-up scans every three days and additional scans as needed if DVT was suspected. The criteria used to diagnose DVT included the presence of a non-compressible vein, evidence of obstruction or filling defects within the vein, absence of respiratory phasicity in the vein segments above the knee, and insufficient flow augmentation in the calf region [13]. The diagnosis of DVT was confirmed by at least two ultrasound specialists.

In this study, patient demographics, comorbidities, and initial laboratory tests at the time of admission were thoroughly examined. The demographic information gathered included details such as age, gender, Body Mass Index (BMI), whether the patient belonged to a minority ethnic group, the duration between injury and hospital admission, how the patient was admitted, the presence of open injuries, the occurrence of multiple injuries, smoking habits, alcohol consumption, and the cause of injury (including car accidents, falls, crush injuries, sprains, and injuries caused by heavy objects). Additionally, patients were assessed using the American Society of Anesthesiologists (ASA) scores, categorizing them into two groups based on their ASA score: either grades 1–2 or grades 3–5. The study also looked into whether surgery was performed and the time elapsed from the injury to when surgery was conducted.

We also included an evaluation of comorbidities such as coronary heart disease, hypertension, diabetes, cerebral infarction, anemia, hypoproteinemia, hypokalemia, and hyponatremia. Furthermore, we analyzed a comprehensive set of laboratory parameters at the time of admission, which encompassed basophils (BAS), eosinophils (EOS), hematocrit (HCT), hemoglobin (HGB), lymphocytes (LYM), mean corpuscular hemoglobin (MCH), monocytes (MON), mean platelet volume (MPV), neutrophils (NEU), platelets (PLT), red blood cells (RBC), white blood cells (WBC), activated partial thromboplastin time (APTT), antithrombin III (ATIII), fibrinogen (FIB), International Normalized Ratio (INR), prothrombin time (PT), prothrombin activity (PTA), thrombin time (TT), albumin (ALB), alkaline phosphatase (ALP), alanine transaminase (ALT), aspartate aminotransferase (AST), calcium (Ca), cholinesterase (CHE), creatine kinase (CK), creatine kinase MB (CKMB), chlorine (Cl), creatinine (CREA), direct bilirubin (DBIL), indirect bilirubin (IBIL), total bilirubin (TBIL), osmotic pressure (OSM), globulin (GLOB), glucose (GLU), lactic dehydrogenase (LDH), potassium (K), sodium (Na), phosphorus (P), magnesium (Mg), total protein (TP), triglycerides (TG), total cholesterol (TC), total carbon dioxide (TCO2), urea (UREA), uric acid (UA). These indicators were collected to provide a detailed health profile of each patient upon their admission.

### Statistics

In our research, we employed the SPSS software (version 25.0, SPSS Inc., New York, USA), setting a p-value of less than 0.05 as the threshold for statistical significance. To assess the distribution of continuous variables, the Shapiro-Wilk test was applied. Variables that followed a normal distribution were described using the mean and standard deviation (SD) and analyzed with the t-test. Conversely, for variables that did not display normality, the Mann-Whitney U test was utilized for statistical comparisons between two groups. Categorical variables were examined using the Chi-square test or Fisher’s exact test, as appropriate, and presented as counts and percentages. Moreover, binary logistic regression analysis was conducted to ascertain the leading indicators of DVT among patients with patellar fractures.

Receiver Operating Characteristic (ROC) analysis was performed to establish the most effective cut-off points for continuous variables like age, based on the maximum Youden index (sensitivity + specificity—1), categorizing them into low or high risk. The diagnostic performance was evaluated through the Area Under the Curve (AUC) of the ROC, which ranges from 0% to 100%, with a larger area indicating superior diagnostic accuracy.

## Result

The research involved a total of 3,311 participants, comprising 2,201 males and 1,110 females. Out of these, 3,032 patients received surgical treatment, while 279 did not undergo surgery. Notably, 30.8% of the patients, accounting for 1,021 individuals, were diagnosed with DVT, while the remainder did not exhibit the condition. In addition, within the group of 1,021 patients diagnosed with DVT, a total of 1,790 clots were identified. Among these patients, 595 had a singular thrombus, while 426 exhibited two or more thrombi in their legs. The most common type of clots observed were in the intermuscular veins, with a total of 772 instances; among these, 516 patients presented with simple intermuscular vein clots alone. In contrast, the least common were clots found in the iliac vein, with a mere 4 occurrences. The analysis indicated that clot formation predominantly occurred in the injured limb, accounting for 96.7% of all clots. Conversely, the uninjured limb was the site of a mere 3.2% of clot occurrences. Further categorization showed a mix of proximal, distal, and mixed-type DVTs, with distal DVTs being the most frequent (82.9%). In addition, in response to identified severe thromboses, 139 patients underwent inferior vena cava filter implantation, although none experienced PE.

Key factors differentiating the DVT and non-DVT groups are outlined in Table 1. Statistically significant differences were observed in age, BMI, presence of open injuries, occurrence of multiple injuries, injury mechanism, surgical treatment, ASA classification, and time from injury to surgery, all with P < 0.001 (except BMI, P = 0.010). No significant differences were found between the two groups in terms of gender, ethnicity, admission pathway, or history of smoking and alcohol use (all P > 0.05).

**Table 1:** Demographics data of patients with and without DVT.

Demographics	DVT group (n = 1021)	Non-DVT group (n = 2290)	*p*
**Age, years**	55.0 (43.0 ~ 65.0)	50.0 (36.0 ~ 60.0)	< 0.001*
**Gender, n (%)**			0.487
Male	670 (65.6%)	1531 (66.9%)	
Female	351 (34.4%)	759 (33.1%)	
**BMI, kg/m2**	25.4 (23.0 ~ 26.1)	25.4 (22.9 ~ 26.2)	0.010*
**Minority Ethnicity, n (%)**			0.451
Yes	73 (7.1%)	181 (7.9%)	
No	948 (92.9%)	2109 (92.1%)	
**Time from injury to admission, hours**	9.0 (5.0 ~ 48.0)	7.3 (3.0 ~ 24.0)	< 0.001*
<12	593 (58.1%)	1417 (61.9%)	0.012*
12–24	165 (16.2%)	390 (17.0%)	
>24	263 (25.8%)	483 (21.1%)	
**Admission Pathway, n (%)**			0.297
Emergency Department	365 (35.7%)	751 (32.8%)	
Outpatient Department	615 (60.2%)	1438 (62.8%)	
Transfer from Other Medical Institutions	2(0.2%)	2 (0.1%)	
Others	39 (3.8%)	99 (4.3%)	
**Open injuries, n (%)**			< 0.001*
Yes	180 (17.6%)	215 (9.4%)	
No	841 (82.4%)	2075 (90.6%)	
**Multiple injuries, n (%)**			< 0.001*
Yes	427 (41.8%)	397 (17.3%)	
No	594 (58.2%)	1893 (82.7%)	
**Mechanism of injury, n (%)**			< 0.001*
Car crash injury	333 (32.6%)	437 (19.1%)	
Fall Injury	612 (59.9%)	1611 (70.3%)	
Crush injury	8 (0.8%)	16 (0.7%)	
Sprain	2 (0.2%)	33 (1.4%)	
Hurt by a heavy object	12 (1.2%)	33 (1.4%)	
Unknown trauma	54 (5.3%)	160 (7.0%)	
**Smoking history**, **n (%)**			0.315
Yes	112 (11.0%)	225 (9.8%)	
No	909 (89.0%)	2065 (90.2%)	
**Alcohol history**, **n (%)**			0.384
Yes	80 (7.8%)	160 (7.0%)	
No	941 (92.2%)	2130 (93.0%)	
**Whether to perform surgery**, **n (%)**			< 0.001*
Yes	966 (94.6%)	2066 (90.2%)	
No	55 (5.4%)	224 (9.8%)	
**ASA classification, n (%)**			< 0.001*
I, II	774 (80.6%)	1839 (90.2%)	
III, IV, V	186 (19.4%)	200 (9.8%)	
**Time from injury to surgery, days**	4.0 (2.0 ~ 8.0)	3.0 (2.0 ~ 5.0)	< 0.001*

BMI = body mass index; ASA = American Society of Anesthesiologists; Values are presented as thenumber (%) or the median (interquartile range)

*p<0.05, statistical significance.

Comorbidity analysis (Table 2) revealed that patients with a history of cerebral infarction (P = 0.027) and hyponatremia (P = 0.019) had an elevated risk of DVT. In contrast, no significant differences were found for other comorbidities such as hypertension, hypokalemia, coronary heart disease, diabetes, hypoproteinemia, and anemia (all P > 0.05).

**Table 2:** Comorbidities data of patients with and without DVT.

Comorbidities	DVT group (n = 1021)	Non-DVT group (n = 2290)	*p*
**Coronary heart disease, n (%)**			0.074
Yes	87 (8.5%)	155 (6.8%)	
No	934 (93.2%)	2135 (93.2%)	
**Hypertension, n (%)**			0.535
Yes	231 (22.6%)	496 (21.7%)	
No	790 (77.4%)	1794 (78.3%)	
**Diabetes, n (%)**			0.058
Yes	93 (9.1%)	259 (11.3%)	
No	928 (90.9%)	2031 (88.7%)	
**Cerebral Infarction, n (%)**			0.027*
Yes	63 (6.2%)	100 (4.4%)	
No	958 (93.8%)	2190 (95.6%)	
**Anemia, n (%)**			0.595
Yes	344 (33.7%)	750 (32.8%)	
No	677 (66.1%)	1540 (67.2%)	
**Hypoproteinemia, n (%)**			0.143
Yes	65 (6.4%)	117 (5.1%)	
No	956 (93.6%)	2173 (94.9%)	
**Hypokalemia, n (%)**			0.927
Yes	126 (12.3%)	280 (12.2%)	
No	895 (87.7%)	2010 (87.7%)	
**Hyponatremia, n (%)**			0.019*
Yes	84 (8.2%)	138 (6.0%)	
No	937 (93.3%)	2152 (94.0%)	

Values are presented as the number (%) or the median (interquartile range)

*p<0.05, statistical significance.

Laboratory findings (Table 3) indicated that the DVT group had significantly higher levels of LYM (P = 0.041) and MCH (P = 0.035), while sodium (P = 0.029) and albumin (P = 0.024) levels were notably lower compared to the non-DVT group. Other laboratory parameters showed no significant differences between the two groups (all P > 0.05).

**Table 3:** Laboratory results of patients with and without DVT.

Laboratory results	DVT group (n = 1021)	Non-DVT group (n = 2290)	*p*
**BAS**	0.03 (0.02 ~ 0.04)	0.03 (0.02 ~ 0.04)	0.110
**EOS**	0.06 (0.02 ~ 0.11)	0.06 (0.02 ~ 0.12)	0.101
**HCT**	37.86 (34.07 ~ 41.10)	38.16 (34.38 ~ 41.53)	0.078
**HGB**	127.00 (113.80 ~ 138.85)	128.00 (115.00 ~ 140.00)	0.160
**LYM**	1.48 (1.13 ~ 1.93)	1.54 (1.18 ~ 1.95)	0.041*
**MCH**	31.17 (30.24 ~ 32.26)	31.09 (29.97 ~ 32.19)	0.035*
**MON**	0.61 (0.48 ~ 0.79)	0.60 (0.46 ~ 0.77)	0.162
**MPV**	8.74 (7.99 ~ 9.51)	8.68 (7.95 ~ 9.53)	0.346
**NEU**	5.84 (4.49 ~ 7.77)	5.80 (4.41 ~ 7.74)	0.763
**PLT**	201.00 (167.05 ~ 245.80)	205.60 (169.57 ~ 245.63)	0.142
**RBC**	4.12 (3.66 ~ 4.46)	4.13 (3.73 ~ 4.50)	0.063
**WBC**	8.18 (6.70 ~ 10.16)	8.17 (6.70 ~ 10.13)	0.939
**APTT**	29.80 (27.40 ~ 32.20)	29.60 (27.40 ~ 32.20)	0.782
**APTT-R**	0.93 (0.85 ~ 1.01)	0.92 (0.85 ~ 1.01)	0.898
**ATIII**	96.94 (87.25 ~ 107.00)	96.94 (87.00 ~ 107.00)	0.783
**D-DIMER**	0.76 (0.30 ~ 1.73)	0.73 (0.30 ~ 1.73)	0.277
**INR**	1.05 (0.99 ~ 1.11)	1.04 (0.99 ~ 1.11)	0.713
**FIB**	3.11 (2.57 ~ 3.84)	3.10 (2.56 ~ 3.73)	0.343
**PT**	11.70 (11.10 ~ 12.60)	11.70 (11.10 ~ 12.60)	0.877
**PTA**	94.00 (85.00 ~ 103.10)	94.00 (86.00 ~ 103.00)	0.640
**TT**	14.40 (13.45 ~ 15.80)	14.50 (13.50 ~ 15.90)	0.247
**ALB**	40.55 (37.60 ~ 43.12)	41.00 (37.93 ~ 43.48)	0.024*
**ALP**	63.00 (51.00 ~ 76.00)	63.00 (51.00 ~ 76.00)	0.625
**ALT**	19.00 (13.00 ~ 31.00)	19.00 (13.00 ~ 32.00)	0.690
**AST**	19.00 (15.00 ~ 29.00)	19.00 (15.00 ~ 29.00)	0.337
**Ca**	2.20 (2.10 ~ 2.30)	2.21 (2.11 ~ 2.31)	0.250
**CHE**	7.64 (6.41 ~ 9.05)	7.77 (6.44 ~ 9.02)	0.333
**CK**	112.20 (69.75 ~ 314.80)	119.25 (71.00 ~ 303.35)	0.799
**CKMB**	13.00 (9.48 ~ 19.25)	12.70 (9.30 ~ 18.32)	0.222
**CL**	104.80 (102.50 ~ 106.60)	104.80 (102.60 ~ 106.90)	0.375
**CREA**	62.50 (54.35 ~ 73.80)	64.00 (54.48 ~ 73.62)	0.681
**DBIL**	4.50 (3.13 ~ 6.43)	4.50 (3.17 ~ 6.30)	0.987
**IBIL**	10.79 (7.50 ~ 14.54)	10.70 (7.75 ~ 14.22)	0.872
**TBIL**	15.20 (11.20 ~ 20.59)	15.05 (11.20 ~ 20.47)	0.871
**OSM**	273,30 (268.90 ~ 282.65)	273.60 (268.80 ~ 281.90)	0.573
**GLOB**	23.45 (20.43 ~ 26.24)	23.00 (20.20 ~ 26.13)	0.304
**GLU**	5.80 (5.18 ~ 6.92)	5.77 (5.15 ~ 6.79)	0.728
**K**	3.90 (3.65 ~ 4.18)	3.91 (3.68 ~ 4.17)	0.340
**LDH**	184.55 (159.42 ~ 229.00)	183.40 (157.00 ~ 226.33)	0.238
**Mg**	0.88 (0.82 ~ 0.95)	0.88 (0.81 ~ 0.94)	0.133
**Na**	139.46 (137.54 ~ 141.40)	139.70 (138.00 ~ 141.60)	0.029*
**P**	1.11 (0.96 ~ 1.25)	1.11 (0.97 ~ 1.26)	0.449
**TC**	4.17 (3.53 ~ 4.82)	4.16 (3.57 ~ 4.84)	0.824
**TCO2**	24.78 (22.41 ~ 27.00)	24.83 (22.60 ~ 27.00)	0.552
**TG**	1.13 (0.80 ~ 1.65)	1.13 (0.81 ~ 1.67)	0.977
**TP**	63.92 (59.63 ~ 67.80)	64.29 (59.78 ~ 68.30)	0.357
**UA**	286.00 (227.50 ~ 357.00)	295.00 (232.00 ~ 364.00)	0.101
**UREA**	5.11 (4.23 ~ 6.23)	5.08 (4.21 ~ 6.23)	0.717

BAS = basophil; EOS = eosinophil; HCT = hematocrit; HGB = hemoglobin; LYM = lymphocyte; MCH = mean corpusular hemoglobin; MON = monocyte; MPV = mean platelet volume; NEU = neutrophil; PLT = platelet; RBC = red blood cell; WBC = white blood cell; APTT = activated partial thromboplastin time; ATIII = antithrombin III; FIB = fibrinoge; INR = International normalized ratio; PT = prothrombin time; PTA = prothrombin activity; TT = thrombin time, ALB = albumin; ALP = alkaline phosphatase; ALT = alanine transaminase; AST = aspartate aminotransferase; Ca = calcium; CHE = cholinesterase; CK = creatine kinase; CKMB = creatine kinase MB; Cl = chlorine; CREA = creatinine; DBIL = direct bilirubin; IBIL = indirect bilirubin; TBIL = total bilirubin; OSM = osmotic pressure; GLOB = globulin; GLU = glucose; LDH = lactic dehydrogenase; K = kalium; Na = sodium; P = phosphorus; Mg = magnesium; TP = total protein; TG = triglyceride; TC = total cholesterol; TCO2 = total carbon dioxide; UREA = ureophil; UA = uric acid; Values are presented as the number (%) or the median (interquartile range)

*p<0.05, statistical significance.

Logistic regression analysis identified several significant predictors for DVT in patellar fracture patients (Table 4). Increasing age (P < 0.001, OR = 1.038, 95% CI: 1.032–1.044), the presence of open injuries (P = 0.002, OR = 1.521, 95% CI: 1.167–1.982), multiple injuries (P < 0.001, OR = 3.623, 95% CI: 2.957–4.438), and the interval from injury to surgery (P < 0.001, OR = 1.097, 95% CI: 1.075–1.121) were all significantly associated with an increased risk of DVT. Conversely, higher levels of albumin (P = 0.029, OR = 0.983, 95% CI: 0.969–0.998) and sodium (P = 0.028, OR = 0.971, 95% CI: 0.946–0.997) were identified as protective factors.

**Table 4:** Binary logistic regression analysis of variables associated with DVT.

Characteristics	OR	95%CI	*p*
**Age**	1.038	1.032 to 1.044	<0.001*
**open injuries**	1.521	1.167 to 1.982	0.002*
**multiple injuries**	3.623	2.957 to 4.438	<0.001*
**ALB**	0.983	0.969 to 0.998	0.029*
**Na**	0.971	0.946 to 0.997	0.028*
**Time from injury to surgery**	1.097	1.075 to 1.121	<0.001*

ALB = albumin; Na = sodium

*p<0.05, statistical significance.

**Fig 2:**
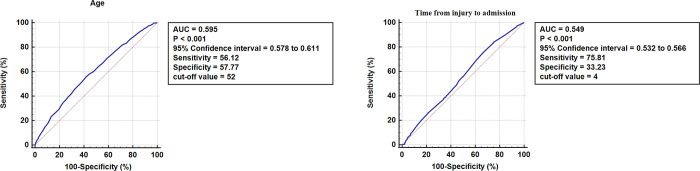
ROC curves for age and time from injury to surgery.

ROC curve analysis (Fig 2) revealed optimal cut-off points for DVT risk factors: 52 years for age and 4 days for the time from injury to surgery. The area under the curve (AUC) for age was 0.595 (P < 0.001, 95% CI: 0.578–0.611), and for the time from injury to surgery, the AUC was 0.549 (P < 0.001, 95% CI: 0.532–0.566).

## Discussion

Patellar fractures, typically caused by direct impacts or forceful quadriceps muscle contractions, can notably impair knee joint functionality. Crucially, the immobilization necessitated by these fractures heightens the risk of DVT due to reduced venous blood flow and increased blood coagulability, triggered by the inflammatory response to injury [5, 6]. DVT emerges as a prevalent issue among hospitalized patients with fractures in the lower limbs, with the potential to escalate into serious conditions such as pulmonary embolism if not adequately managed. Studies reveal that the incidence of DVT among orthopedic surgery patients is influenced by various factors [12, 13]. However, research in this area remains relatively limited, highlighting the need for further investigation. Our study advances beyond prior research by conducting a comprehensive, large-scale, multicenter investigation that quantifies the incidence rates of thrombosis in patients with patellar fractures. Subsequently, it delves into examining the risk factors for DVT, alongside exploring the types of thrombi prevalent in this specific patient cohort, thereby providing a fuller picture of the thrombotic risks associated with patellar fractures.

Our research revealed that during their hospital stay, 30.8% of patients with patellar fractures developed DVT, with a total of 1790 thrombi detected among 1021 patients. Breaking this down further, we observed 16 patients (1.6%) with proximal DVT, 846 patients (82.9%) with distal DVT, and 159 patients (15.5%) with mixed DVT. This starkly contrasts with lower incidence rates reported in other studies, such as Tan et al.’s 4.4% (35 out of 790) and Yang et al.’s 5.8% (48 out of 827) [11, 14, 15]. The significant disparities observed can be attributed to two primary reasons. Firstly, the aforementioned studies solely concentrated on identifying risk factors associated with thrombus formation pre- or post-surgery in patients with patellar fractures, rather than investigating the development of DVT throughout the entirety of the hospital stay. More critically, these studies all neglected the analysis of intermuscular vein thrombosis—a frequently overlooked yet prevalent form of DVT in this patient population—resulting in their reported incidence rates being comparatively lower. Notably, the literature indicates even "mild" forms of DVT, like intermuscular vein thrombosis, carry significant PE risks if left untreated, underscoring the importance of not overlooking even the clinically mildest forms of thrombosis [16]. Our findings echo Zhang et al.’s analysis, which reported a higher DVT incidence of 52.5% by incorporating the often-missed factor of intermuscular vein thrombosis [17]. Besides, our findings revealed that DVTs manifested not only in the limb impacted by injury (with 1,731 clots, representing 96.7%, found on the injured side) but also in the unaffected limb, where 59 clots (3.2%) were detected. This observation aligns with the conclusions drawn by Wang et al., highlighting a significant post-fracture risk of thrombosis across both limbs. The cause is twofold: a hypercoagulable state induced by the fracture and decreased mobility from prolonged bed rest. Consequently, this emphasizes the critical need for comprehensive DVT screenings, encompassing both the injured and non-injured extremities to prevent overlooked diagnoses [18].

Our logistic regression analysis revealed that the risk of developing DVT in patients with patellar fractures increases with age, with each additional year increasing the risk by 1.038 times, and identifies 52 years as the critical cut-off point, beyond which patients are considered high risk. This is consistent with previous studies [19], such as those by Chang et al., who analyzed 11,891 patients with closed lower extremity fractures and identified age as an independent risk factor for DVT complications [20], and Tan et al., who linked age closely with the occurrence of preoperative DVT in similar patients [11]. Goel et al. also recognized that patients over 40 are at increased risk for DVT following fractures below the knee [21]. The greater vulnerability of older patients can be attributed to several age-related physiological changes: reduced mobility which slows blood flow in the lower extremities, enhancing clot formation risk [8]; increased blood coagulability, making older individuals more prone to clots, especially post-injury or surgery [6]; and vascular changes, such as alterations in the endothelial lining of blood vessels, which predispose to clot formation and impair blood flow, thereby elevating DVT risk [22]. Given these insights, it is imperative for clinicians to implement customized DVT prevention strategies for older adults, including early mobility protocols, enhanced coagulation monitoring, and potentially more aggressive anticoagulation treatments for those aged 52 and above, to effectively manage and mitigate their increased risk.

Our research establishes a clear link between surgical delays and an increased incidence of DVT in patients with patellar fractures. We found that each day’s delay in surgery amplifies the risk of developing DVT by 1.097 times, with risks escalating significantly when surgery is postponed beyond four days. Factors contributing to surgical delays often include limited availability of surgical teams or operating rooms, especially during peak times when hospital resources are stretched thin [8]. Furthermore, the complexity involved in diagnosing the exact nature of the fracture or assessing associated injuries may require additional diagnostic procedures, further postponing surgery [4]. Delays are sometimes inevitable when patients develop complicating medical conditions such as fever, severe anemia, or significant thrombotic complications during the waiting period, all requiring stabilization before surgery can proceed [7]. It is noteworthy that in our study of 1021 patients, 32 experienced proximal DVT before their scheduled surgery. These cases of preoperative DVT inevitably led to further delays. To minimize confounding variables and ensure the accuracy of our findings, we excluded this subgroup from our analysis. Geerts et al. have indicated that immobility and prolonged preoperative periods contribute to the elevated risk of DVT [23]. Other researchers have also identified a correlation between surgical delays and increased rates of DVT, underscoring the importance of early intervention [24, 25]. These findings align with ours. Surgical delays in patients with lower limb fractures can increase the risk of DVT, primarily due to prolonged immobility, which promotes venous stasis. This immobility impedes the normal muscle-pump action of the legs, leading to blood pooling and potential clot formation. Additionally, injury-related inflammation can increase the blood’s propensity to clot, further elevating DVT risk when surgery is delayed [26–28].

In this study, we define open and multiple injuries as open wounds and/or injuries at other sites on the same limb as a patellar fracture, typically resulting from high-energy trauma such as traffic accidents or significant falls. We found that both open and multiple injuries significantly increase the risk of DVT in patients with patellar fractures, with incidences increasing by 1.521 and 3.623 times, respectively. Consistent with findings from Shi et al., who identified multiple injuries as independent risk factors for DVT in patients with lower-extremity fractures in emergency intensive care settings, and Liu et al., who noted a higher risk of DVT in patients with open injuries compared to those with closed injuries, our results underscore the correlation between injury severity and DVT occurrence [29, 30]. The external forces causing these injuries often lead to more severe vascular intima damage, blood stasis, and coagulation disorders. Furthermore, patients with these types of injuries are likely to experience hemorrhagic shock, hypoperfusion, and ischemia-reperfusion injuries after fluid resuscitation, exacerbating the injury severity and increasing DVT risk [27]. Besides, inflammation triggered by open and multiple injuries prompts the release of procoagulant and antifibrinolytic factors, enhancing blood coagulation. The severe soft tissue damage associated with these injuries also releases tissue factor, a potent activator of the clotting process. Moreover, these injuries frequently result in disrupted or slowed local blood flow, compounding the risk of DVT development [30–32]. Therefore, it is crucial for physicians treating patients with patellar fractures accompanied by multiple and open injuries to increase their vigilance and proactivity in thrombosis management.

In our study, elevated levels of ALB and sodium have been identified as protective factors against the development of DVT in patients with patellar fractures. Specifically, we found that each 1 g/L increase in albumin levels is associated with a 1.7% decrease in the risk of DVT (P = 0.029), and each 1 mmol/L increase in sodium levels reduces the risk by 2.9% (P = 0.028). ALB, a major plasma protein, a major plasma protein, plays a pivotal role in maintaining oncotic pressure and regulating fluid distribution within the vascular and interstitial spaces. This function is crucial in minimizing blood viscosity, which, in turn, reduces thrombosis risk. Lower levels of ALB often indicate underlying conditions such as malnutrition or an inflammatory state, both of which are recognized risk factors for DVT [6]. Similarly, sodium, the primary cation in extracellular fluid, is essential for maintaining fluid volume and vascular stability. Lower sodium concentrations may reflect systemic adjustments following blood loss related to knee trauma and suggest increased blood hypercoagulability resulting from the initial trauma and fracture severity. Previous research supports the association between low sodium levels and the development of DVT [33]. Our findings further validate these observations from an additional perspective. Therefore, monitoring and adjusting albumin and sodium levels in patients with patellar fractures could be crucial in preventing DVT. Clinically, ensuring optimal nutritional status and electrolyte balance should form a fundamental part of comprehensive DVT prophylaxis.

Despite the comprehensive analysis and robust methodology of our study, several limitations warrant mention. Firstly, the retrospective nature of the study constrains our ability to establish causality. Prospective studies would be better suited to control variables not accounted for in a retrospective setting and provide more definitive evidence. Secondly, while logistic regression was employed to account for a variety of confounders, the possibility of unmeasured or residual confounding remains. Factors such as the classification of fractures and the completeness of medical records might have influenced the results.

Thirdly, by excluding patients with a history of DVT or thromboembolic events, as well as those on chronic anticoagulation therapy, we might have underestimated the prevalence and significance of potential risk factors for DVT. This selection bias could limit the generalizability of the study’s outcomes to the broader population. Furthermore, excluding patients with prior DVT, coagulation disorders, or anticoagulant use may introduce additional selection bias, potentially underestimating DVT incidence in the general population.

Most importantly, in our study, we identified the time from injury to surgery as a significant risk factor for the development of DVT in patients with patellar fractures. This means that we assumed preoperative DVT would not affect the conduct of the surgery. To minimize bias, we strictly excluded patients whose surgeries were delayed due to severe preoperative thrombosis. However, the possibility of residual confounding by undetected preoperative DVT remains, which could exaggerate the association between surgical delay and increased DVT risk.

Last but not least, the cut-off points for age (52 years) and surgical delay (4 days) showed relatively low predictive accuracy, with AUC values of 0.595 and 0.549, respectively. While these factors are significant, their low AUC suggests they may not be reliable predictors of DVT on their own. This could be due to the complex nature of DVT, which may not be fully captured by these two variables. Nonetheless, age and surgical delay remain important risk factors in patellar fracture patients. Future research should focus on refining these thresholds and incorporating additional risk factors to enhance prediction accuracy.

Another limitation is the lack of long-term follow-up. DVT can develop or progress over time, and without extended follow-up, we are unable to capture the full spectrum of DVT risk and its long-term consequences. Future studies with longer follow-up periods would be valuable in assessing the long-term incidence of DVT in patellar fracture patients and refining risk prediction models.

In conclusion, our study reveals a high incidence of DVT in patients with patellar fractures, especially when intermuscular vein thrombosis is included, and highlights the critical importance of timely surgical interventions and rigorous preoperative screening to manage risk factors such as prolonged time from injury to surgery, presence of open and multiple injuries, and advanced age, while also noting that elevated levels of albumin and sodium serve as protective factors. Our study advances understanding of DVT risk factors in patellar fracture patients, providing valuable insights for optimizing clinical protocols and improving patient care.

## Abbreviations

DVT: deep vein thrombosis
PE: pulmonary embolism
LMWH: low-molecular-weight heparin sodium
DUS: Doppler Ultrasound Scans
BMI: Body Mass Index
ASA: American Society of Anesthesiologists
BAS: basophils
EOS: eosinophils
HCT: hematocrit
HGB: hemoglobin
LYM: lymphocytes
MCH: mean corpuscular hemoglobin
MON: monocytes
MPV: mean platelet volume
NEU: neutrophils
PLT: platelets
RBC: red blood cells
WBC: white blood cells
APTT: activated partial thromboplastin time
ATIII: antithrombin III
FIB: fibrinogen
INR: International NormalizedRatio
PT: prothrombin time
PTA: prothrombin activity
TT: thrombin time
ALB: albumin
ALP: alkaline phosphatase
ALT: alanine transaminase
AST: aspartate aminotransferase
Ca: calcium
CHE: cholinesterase
CK: creatine kinase
CKMB: creatine kinase MB
Cl: chlorine
CREA: creatinine
DBIL: direct bilirubin
IBIL: indirect bilirubin
TBIL: total bilirubin
OSM: osmotic pressure
GLOB: globulin
GLU: glucose
LDH: lactic dehydrogenase
K: potassium
Na: sodium
P: phosphorus
Mg: magnesium
TP: total protein
TG: triglycerides
TC: total cholesterol
TCO2: total carbon dioxide
UREA: urea
UA: uric acid
ROC: Receiver Operating Characteristic
AUC: Area Under the Curve
